# Supplementary material for: γδ T cell-derived IL-4 initiates CD8+ T cell immunity
Source: Nat Immunol. 2026 Jan 30;27(2):295–307. doi: 10.1038/s41590-025-02397-z (PMC12864030; doi:10.1038/s41590-025-02397-z)
Supplement: Supplementary file 1 — Reporting Summary [file 41590_2025_2397_MOESM1_ESM.pdf]

nature portfolio

Corresponding author(s): Lynette Beattie

Last updated by author(s): 25/11/25

Reporting Summary

Nature Portfolio wishes to improve the reproducibility of the work that we publish. This form provides structure for consistency and transparency in reporting. For further information on Nature Portfolio policies, see our Editorial Policies and the Editorial Policy Checklist.

Statistics

For all statistical analyses, confirm that the following items are present in the figure legend, table legend, main text, or Methods section.

n/a	Confirmed	
☐	☒	The exact sample size (*n*) for each experimental group/condition, given as a discrete number and unit of measurement
☐	☒	A statement on whether measurements were taken from distinct samples or whether the same sample was measured repeatedly
☐	☒	The statistical test(s) used AND whether they are one- or two-sided<br>*Only common tests should be described solely by name; describe more complex techniques in the Methods section.*
☒	☐	A description of all covariates tested
☐	☒	A description of any assumptions or corrections, such as tests of normality and adjustment for multiple comparisons
☐	☒	A full description of the statistical parameters including central tendency (e.g. means) or other basic estimates (e.g. regression coefficient) AND variation (e.g. standard deviation) or associated estimates of uncertainty (e.g. confidence intervals)
☐	☒	For null hypothesis testing, the test statistic (e.g. F, t, r) with confidence intervals, effect sizes, degrees of freedom and P value noted<br>*Give P values as exact values whenever suitable.*
☒	☐	For Bayesian analysis, information on the choice of priors and Markov chain Monte Carlo settings
☒	☐	For hierarchical and complex designs, identification of the appropriate level for tests and full reporting of outcomes
☐	☒	Estimates of effect sizes (e.g. Cohen's *d*, Pearson's *r*), indicating how they were calculated

Our web collection on statistics for biologists contains articles on many of the points above.

Software and code

Policy information about availability of computer code

Data collection	For flow cytometry experiments, cells were acquired on a Cytek Aurora using the SpectroFlo v3.0 system (Cytek) as stated in the relevant Methods sections. Cells were sorted using a BD FACSAriaTM III Cell Sorter and the BD FACSDiva program v9.
Data analysis	For RNA sequencing analysis: STAR (version 2.7.8a), featureCounts (version 2.0.0), R (version 4.5.0), EDASeq (version 2.42.0), ruv (version 0.9.7.1), edgeR (version 4.6.2), limma (version 3.64.1), ggplot2 (version 3.5.2), ggrepel (version 0.9.6), ggvenn (version 0.1.10), pheatmap (version 1.0.13).<br>Analyses of flow cytometry data were performed on FlowJo v10.10.0. Statistical analyses of graphed data were performed using GraphPad Prism software V10.<br>Cells were sorted using a BD FACSAriaTM III Cell Sorter and the BD FACSDiva program v9.<br>These packages are stated in the relevant Methods sections.

For manuscripts utilizing custom algorithms or software that are central to the research but not yet described in published literature, software must be made available to editors and reviewers. We strongly encourage code deposition in a community repository (e.g. GitHub). See the Nature Portfolio guidelines for submitting code & software for further information.

Data

Policy information about availability of data

All manuscripts must include a data availability statement. This statement should provide the following information, where applicable:

- Accession codes, unique identifiers, or web links for publicly available datasets
- A description of any restrictions on data availability
- For clinical datasets or third party data, please ensure that the statement adheres to our policy

Data Availability
Mouse RNA sequencing data are deposited with EMBL-EBI gene expression database accession number E-MTAB-16291 (request currently pending).
All other raw data is provided as Source Data Files and/or are available from the corresponding author upon reasonable request.

Research involving human participants, their data, or biological material

Policy information about studies with human participants or human data. See also policy information about sex, gender (identity/presentation), and sexual orientation and race, ethnicity and racism.

Reporting on sex and gender	Reported in 'Supplementary Table 1: Human cohort clinical characteristics'
Reporting on race, ethnicity, or other socially relevant groupings	Numbers of males and females used in the study are described in Supplementary Table 1. The data presented are not disaggregated by gender.<br>The samples used in this study were recruited as part of a different study as published in https://doi.org/10.1016/S0140-6736(07)60160-3
Population characteristics	Reported in: Supplementary Table 1: Human cohort clinical characteristics
Recruitment	Patients were recruited as part of the original study described in: https://doi.org/10.1016/S0140-6736(07)60160-3<br>Patients presenting to study hospitals with microscopy-diagnosed malaria of any species were enrolled as a part of their treatment following written informed consent. Healthy and Endemic controls enrolled following written informed consent.
Ethics oversight	Studies in natural infection settings of Timika in Papua were approved by the ethics committees of the Northern Territory Department of Health and Menzies School of Health Research (HREC 05-16, HREC 03-64, HREC 10-13970), the QIMR Berghofer Medical Research Institute Human Ethics Committee (HREC P3444) and the Indonesian National Institute of Health Research and Development (NIHRD KS.02.01.2.1.4042).

Note that full information on the approval of the study protocol must also be provided in the manuscript.

Field-specific reporting

Please select the one below that is the best fit for your research. If you are not sure, read the appropriate sections before making your selection.

☒ Life sciences ☐ Behavioural & social sciences ☐ Ecological, evolutionary & environmental sciences

For a reference copy of the document with all sections, see nature.com/documents/nr-reporting-summary-flat.pdf

Life sciences study design

All studies must disclose on these points even when the disclosure is negative.

Sample size	Required sample sizes were estimated based on the average magnitude of variation in previous or pilot experiments, constrained by the availability of biological samples, particularly radiation attenuated sporozoites, which are a limiting factor for numbers of animals used in most experiments contained in this study. Generally, 3-8 mice were used per group and then experiments independently repeated at least 2-3 times and results pooled.
Data exclusions	Data were analysed without exclusion unless explicitly stated.
Replication	All data are representative of multiple independent experiments repeated at least 2-3 times.
Randomization	Age/Sex matched mice were randomly allocated into groups.
Blinding	Investigators were not blinded.

Behavioural & social sciences study design

All studies must disclose on these points even when the disclosure is negative.

Study description	*Briefly describe the study type including whether data are quantitative, qualitative, or mixed-methods (e.g. qualitative cross-sectional, quantitative experimental, mixed-methods case study).*
Research sample	*State the research sample (e.g. Harvard university undergraduates, villagers in rural India) and provide relevant demographic information (e.g. age, sex) and indicate whether the sample is representative. Provide a rationale for the study sample chosen. For studies involving existing datasets, please describe the dataset and source.*
Sampling strategy	*Describe the sampling procedure (e.g. random, snowball, stratified, convenience). Describe the statistical methods that were used to predetermine sample size OR if no sample-size calculation was performed, describe how sample sizes were chosen and provide a rationale for why these sample sizes are sufficient. For qualitative data, please indicate whether data saturation was considered, and what criteria were used to decide that no further sampling was needed.*
Data collection	*Provide details about the data collection procedure, including the instruments or devices used to record the data (e.g. pen and paper, computer, eye tracker, video or audio equipment) whether anyone was present besides the participant(s) and the researcher, and whether the researcher was blind to experimental condition and/or the study hypothesis during data collection.*
Timing	*Indicate the start and stop dates of data collection. If there is a gap between collection periods, state the dates for each sample cohort.*
Data exclusions	*If no data were excluded from the analyses, state so OR if data were excluded, provide the exact number of exclusions and the rationale behind them, indicating whether exclusion criteria were pre-established.*
Non-participation	*State how many participants dropped out/declined participation and the reason(s) given OR provide response rate OR state that no participants dropped out/declined participation.*
Randomization	*If participants were not allocated into experimental groups, state so OR describe how participants were allocated to groups, and if allocation was not random, describe how covariates were controlled.*

Ecological, evolutionary & environmental sciences study design

All studies must disclose on these points even when the disclosure is negative.

Study description	*Briefly describe the study. For quantitative data include treatment factors and interactions, design structure (e.g. factorial, nested, hierarchical), nature and number of experimental units and replicates.*
Research sample	*Describe the research sample (e.g. a group of tagged Passer domesticus, all Stenocereus thurberi within Organ Pipe Cactus National Monument), and provide a rationale for the sample choice. When relevant, describe the organism taxa, source, sex, age range and any manipulations. State what population the sample is meant to represent when applicable. For studies involving existing datasets, describe the data and its source.*
Sampling strategy	*Note the sampling procedure. Describe the statistical methods that were used to predetermine sample size OR if no sample-size calculation was performed, describe how sample sizes were chosen and provide a rationale for why these sample sizes are sufficient.*
Data collection	*Describe the data collection procedure, including who recorded the data and how.*
Timing and spatial scale	*Indicate the start and stop dates of data collection, noting the frequency and periodicity of sampling and providing a rationale for these choices. If there is a gap between collection periods, state the dates for each sample cohort. Specify the spatial scale from which the data are taken*
Data exclusions	*If no data were excluded from the analyses, state so OR if data were excluded, describe the exclusions and the rationale behind them, indicating whether exclusion criteria were pre-established.*
Reproducibility	*Describe the measures taken to verify the reproducibility of experimental findings. For each experiment, note whether any attempts to repeat the experiment failed OR state that all attempts to repeat the experiment were successful.*
Randomization	*Describe how samples/organisms/participants were allocated into groups. If allocation was not random, describe how covariates were controlled. If this is not relevant to your study, explain why.*
Blinding	*Describe the extent of blinding used during data acquisition and analysis. If blinding was not possible, describe why OR explain why blinding was not relevant to your study.*

Did the study involve field work? ☐ Yes ☐ No

Field work, collection and transport

Field conditions	*Describe the study conditions for field work, providing relevant parameters (e.g. temperature, rainfall).*
Location	*State the location of the sampling or experiment, providing relevant parameters (e.g. latitude and longitude, elevation, water depth).*
Access & import/export	*Describe the efforts you have made to access habitats and to collect and import/export your samples in a responsible manner and in compliance with local, national and international laws, noting any permits that were obtained (give the name of the issuing authority, the date of issue, and any identifying information).*
Disturbance	*Describe any disturbance caused by the study and how it was minimized.*

Reporting for specific materials, systems and methods

We require information from authors about some types of materials, experimental systems and methods used in many studies. Here, indicate whether each material, system or method listed is relevant to your study. If you are not sure if a list item applies to your research, read the appropriate section before selecting a response.

Materials & experimental systems

n/a	Involved in the study	
☐	☒	Antibodies
☒	☐	Eukaryotic cell lines
☒	☐	Palaeontology and archaeology
☐	☒	Animals and other organisms
☐	☒	Clinical data
☒	☐	Dual use research of concern
☒	☐	Plants

Methods

n/a	Involved in the study	
☒	☐	ChIP-seq
☐	☒	Flow cytometry
☒	☐	MRI-based neuroimaging

Antibodies

Antibodies used

Name, Fluorochrome, Supplier/Manufacturer, Catalog #, dilution
For human studies:
LiveDead Blue - Invitrogen L34962 (1:5000)
Vδ2 APC/Fire 750 B6 Biolegend 331420 (1:100)
CD3 BUV805 SK7 BD Biosciences 612893 (1:200)
CCR7 PerCP-Cy5.5 150503 BD Biosciences 561144 (2:25)
CXCR5 (CD185) BV711 J252D4 Biolegend 356934 (1:50)
PD-1 (CD279) PE-Cy7 EH12.1 BD Biosciences 561272 (1:50)
IFNγ BUV395 B27 BD Biosciences 563563 (1:50)
TNF BV750 Mab11 BD Biosciences 566359 (1:100)
IL4 APC MP4-25D2 Biolegend 500812 (1:10)
CD14 BV510 M5E2 Biolegend 301842 (1:50)
CD19 BV510 SJ25D1 Biolegend 363020 (1:50)
For Mouse studies:
LIVE/DEAD Fixable Near IR Dead Cell Stain Kit Invitrogen L10119 (1:1000)
B220 BUV496 RA3-6B2 BD Biosciences 356938 (1:500)
CD11a PE-Cy7 2D7 BD Biosciences 558191 (1:500)
CD11b BV711 M1/70 Biolegend 558191 (1:300)
CD11b BUV661 M1/70 BD Biosciences 612977 (1:300)
CD11c BV605 N418 Biolegend 117333 (1:200)
CD19 PerCP-Cy5.5 1D3 Biolegend 152406 (1:400)
CD124 (IL-4Ra) PE mIL4R-M1 BD Biosciences 552509 (1:200)
CD124 (IL-4Ra) PE-Cy7 11B11 Biolegend 504117 (1:200)
CD172a (SIRPa) FITC P84 Biolegend 144006 (1:200)
CD212 (IL-12Rb1) PE 114 BD Biosciences 551974 (1:100)
CD24 PE M1/69 BD Biosciences 553262 (1:200)
CD25 BV605 PC61 BD Biosciences 563061 (1:200)
CD27 BUV737 LG.3A10 BD Biosciences 612831 (1:200)
CD4 BUV395 RM4.5 BD Biosciences 565974 (1:400)
CD44 APC-R700 IM7 Biolegend 565480 (1:300)
CD62L BV605 Mel-14 BD Biosciences 563252 (1:200)
CD62L PerCP-Cy5.5 Mel-14 Biolegend 104432 (1:200)
CD69 PE-Cy5 H1.2D3 Biolegend 104432 (1:200)
CD8a BV711 53-6.7 Biolegend 100748 (1:400)
CD8a FITC 53-6.7 BD Biosciences 553031 (1:400)

CD8a RB744 53-6.7 BD Biosciences 570486 (1:400)
MHC-II (I-A/E) Alexa Fluor 700 M5/114.15.2 Invitrogen 56-5321-82 (1:400)
NK1.1 BUV563 PK136 BD Biosciences 741233 (1:400)
NK1.1 PerCP-Cy5.5 PK136 BD Biosciences 551114 (1:400)
Streptavidin PE - Invitrogen S866 (1.5uL/ug of monomer)
TCRb BV510 H57-597 BD Biosciences 563221 (1:400)
TCRb BV786 H57-597 BD Biosciences 568222 (1:400)
TCRb BUV805 H57-597 BD Biosciences 748405 (1:400)
TCRb PerCP-Cy5.5 H57-597 Biolegend 109227 (1:400)
TCRd BV421 GL3 BD Biosciences 562892 (1:200)
TCRd BV605 GL3 Biolegend 118129 (1:200)
Va8.3 PE T50 Biolegend 125707 (1:300)
Vb12 PE Mr11-1 Biolegend 139704 (1:300)
TCR Vg1 PE 2.11 Biolegend 141105 (1:300)
XCR1 BV785 ZET Biolegend 148225 (1:200)
IFNg BV480 XMG1.2 BD Biosciences 566097 (1:200)
IL-4 Alexa Fluor 647 11B11 BD Biosciences 557739 (1:75)
Tbet BV421 4B10 Biolegend 644832 (1:200)
Tbet RB613 4B10 BD Biosciences 571286 (1:200)
GATA3 PE-Cy7 L50-823 BD Biosciences 560405 (1:50)
CXCR5 PE-Dazzle594 L138D7 Biolegend 145522 (1:200)
PD-1 PE-Fire810 29F.1A12 Biolegend 135253 (1:200)
CD25 APC PC61.5 Invitrogen 17-0251-81 (1:200)
B220 n/a RA3-6B2 Invitrogen 14-0452-85 (1:500)

Validation

All antibodies are from commercial sources and have been validated by the vendors. Validation data are available on the manufacture's websites.
Antibodies were titrated by the authors to find the optimal dilution (amount) for staining.

Eukaryotic cell lines

Policy information about cell lines and Sex and Gender in Research

Cell line source(s)

State the source of each cell line used and the sex of all primary cell lines and cells derived from human participants or vertebrate models.

Authentication

Describe the authentication procedures for each cell line used OR declare that none of the cell lines used were authenticated.

Mycoplasma contamination

Confirm that all cell lines tested negative for mycoplasma contamination OR describe the results of the testing for mycoplasma contamination OR declare that the cell lines were not tested for mycoplasma contamination.

Commonly misidentified lines
(See ICLAC register)

Name any commonly misidentified cell lines used in the study and provide a rationale for their use.

Palaeontology and Archaeology

Specimen provenance

Provide provenance information for specimens and describe permits that were obtained for the work (including the name of the issuing authority, the date of issue, and any identifying information). Permits should encompass collection and, where applicable, export.

Specimen deposition

Indicate where the specimens have been deposited to permit free access by other researchers.

Dating methods

If new dates are provided, describe how they were obtained (e.g. collection, storage, sample pretreatment and measurement), where they were obtained (i.e. lab name), the calibration program and the protocol for quality assurance OR state that no new dates are provided.

☐ Tick this box to confirm that the raw and calibrated dates are available in the paper or in Supplementary Information.

Ethics oversight

Identify the organization(s) that approved or provided guidance on the study protocol, OR state that no ethical approval or guidance was required and explain why not.

Note that full information on the approval of the study protocol must also be provided in the manuscript.

Animals and other research organisms

Policy information about studies involving animals; ARRIVE guidelines recommended for reporting animal research, and Sex and Gender in Research

Laboratory animals

C57BL/6, B6.SJL-PtprcaPep3b/BoyJ (CD45.1), PbT-I GFP, PbT-II GFP, Tcrd-/-, Trdctm1(EGFP/HBEGF/luc)Impr (TCRd-GDL), Il4-/-, Batf3-/-, Cd40l (Cd154)-/- , Il4ra-/-, 4C13R, Ifng-/-, Tcra-/-, Il12p40-/- mice were used as detailed in Methods under 'Mice'. All experimental mice were used at 6-12 weeks of age, or up to 20 weeks for chimeras. Mice were bred and maintained at the Peter

	Doherty Institute for Infection and Immunity mouse facility and housed at 20–26°C, 45–65% humidity on a 12-h day–night light cycle. Mice used for parasite generation were were purchased from the Monash Animal Service and held at the School of Botany, The University of Melbourne. All mice were maintained on a standard chow diet.
Wild animals	No wild animals were used.
Reporting on sex	Both male and female mice were used as Detailed in Methods under 'Mice'. All mice were age and sex matched within experiments.
Field-collected samples	No field-collected samples were used in this study.
Ethics oversight	Stated in the methods section under 'Mice.' All experimental work and animal handling was conducted in strict accordance with the standards approved by the Animal Ethics Committee at the University of Melbourne (ethic project ID: 2015168, 20088, 27552).

Note that full information on the approval of the study protocol must also be provided in the manuscript.

Clinical data

Policy information about clinical studies

All manuscripts should comply with the ICMJE guidelines for publication of clinical research and a completed CONSORT checklist must be included with all submissions.

Clinical trial registration	This was not a clinical trial
Study protocol	PBMCs and plasma samples were collected during previously conducted trials in Timika, Papua, between 2004 and 2005. Timika is a lowland town located in the South-Central Papuan province of Indonesia.
Data collection	Malaria transmission is perennial in lowland Papua, with a prevalence of 28.3% in children under five years, 46.3% in children aged 5-15 years, and 36.8% in adults over 15 years. For the parent trials, adults with slide-confirmed malaria and fever, or a history of fever within the last 48 hours, were enrolled in randomised controlled trials of artemisinin combined therapy. Malaria parasite infection was categorised as P. falciparum or P. vivax mono-infection via microscopy. Exclusion criteria included pregnant or lactating women and children with a body weight of 10kg and under. In a subset of trial participants, blood samples were collected at enrolment for PBMC isolation (<20 mL). For the current study, PBMCs were selected from patients with parasite infection categorised as P. falciparum mono-infection via microscopy.
Outcomes	The primary outcome data was described in parent collection study (https://www.sciencedirect.com/science/article/pii/S0140673607601603?via%3Dihub).<br>Here, the secondary outcome is to investigate the immune cell responses involved in the pathogenesis of Plasmodium falciparum infection as potential targets for immunotherapeutic interventions

Dual use research of concern

Policy information about dual use research of concern

Hazards

Could the accidental, deliberate or reckless misuse of agents or technologies generated in the work, or the application of information presented in the manuscript, pose a threat to:

No	Yes	
☐	☐	Public health
☐	☐	National security
☐	☐	Crops and/or livestock
☐	☐	Ecosystems
☐	☐	Any other significant area

Experiments of concern

Does the work involve any of these experiments of concern:

No	Yes	
☐	☐	Demonstrate how to render a vaccine ineffective
☐	☐	Confer resistance to therapeutically useful antibiotics or antiviral agents
☐	☐	Enhance the virulence of a pathogen or render a nonpathogen virulent
☐	☐	Increase transmissibility of a pathogen
☐	☐	Alter the host range of a pathogen
☐	☐	Enable evasion of diagnostic/detection modalities
☐	☐	Enable the weaponization of a biological agent or toxin
☐	☐	Any other potentially harmful combination of experiments and agents

Plants

Seed stocks	*Report on the source of all seed stocks or other plant material used. If applicable, state the seed stock centre and catalogue number. If plant specimens were collected from the field, describe the collection location, date and sampling procedures.*
Novel plant genotypes	*Describe the methods by which all novel plant genotypes were produced. This includes those generated by transgenic approaches, gene editing, chemical/radiation-based mutagenesis and hybridization. For transgenic lines, describe the transformation method, the number of independent lines analyzed and the generation upon which experiments were performed. For gene-edited lines, describe the editor used, the endogenous sequence targeted for editing, the targeting guide RNA sequence (if applicable) and how the editor was applied.*
Authentication	*Describe any authentication procedures for each seed stock used or novel genotype generated. Describe any experiments used to assess the effect of a mutation and, where applicable, how potential secondary effects (e.g. second site T-DNA insertions, mosiacism, off-target gene editing) were examined.*

ChIP-seq

Data deposition

☐ Confirm that both raw and final processed data have been deposited in a public database such as GEO.

☐ Confirm that you have deposited or provided access to graph files (e.g. BED files) for the called peaks.

Data access links<br>*May remain private before publication.*	*For "Initial submission" or "Revised version" documents, provide reviewer access links. For your "Final submission" document, provide a link to the deposited data.*
Files in database submission	*Provide a list of all files available in the database submission.*
Genome browser session<br>(e.g. UCSC)	*Provide a link to an anonymized genome browser session for "Initial submission" and "Revised version" documents only, to enable peer review. Write "no longer applicable" for "Final submission" documents.*

Methodology

Replicates	*Describe the experimental replicates, specifying number, type and replicate agreement.*
Sequencing depth	*Describe the sequencing depth for each experiment, providing the total number of reads, uniquely mapped reads, length of reads and whether they were paired- or single-end.*
Antibodies	*Describe the antibodies used for the ChIP-seq experiments; as applicable, provide supplier name, catalog number, clone name, and lot number.*
Peak calling parameters	*Specify the command line program and parameters used for read mapping and peak calling, including the ChIP, control and index files used.*
Data quality	*Describe the methods used to ensure data quality in full detail, including how many peaks are at FDR 5% and above 5-fold enrichment.*
Software	*Describe the software used to collect and analyze the ChIP-seq data. For custom code that has been deposited into a community repository, provide accession details.*

Flow Cytometry

Plots

Confirm that:

☒ The axis labels state the marker and fluorochrome used (e.g. CD4-FITC).

☒ The axis scales are clearly visible. Include numbers along axes only for bottom left plot of group (a 'group' is an analysis of identical markers).

☒ All plots are contour plots with outliers or pseudocolor plots.

☒ A numerical value for number of cells or percentage (with statistics) is provided.

Methodology

Sample preparation	Described in the Methods section
Instrument	Cells were acquired on a Cytek Aurora using the SpectroFlo v3.0 system (Cytek) and further analysed on FlowJo (TreeStar, Inc)
Software	SpectroFlo v3.0 system (Cytek) and further analysed on FlowJo (TreeStar, Inc)
Cell population abundance	Absolute numbers of cells per organ are shown in most instances, with gating strategies described or shown. Cell sort purities for Figure 6 were above 95% pure (of live cells).
Gating strategy	Gating strategies are described in the text or the figure legends or gating strategies are shown. Typically, lymphocytes are first gated using FSC-A vs SSC-A, followed by doublet exclusion using FSC-A/H. Dead cells were then excluded by Live-Dead Blue or NIR staining followed by specific gating per experiment to detect target cells.

☒ Tick this box to confirm that a figure exemplifying the gating strategy is provided in the Supplementary Information.

Magnetic resonance imaging

Experimental design

Design type	*Indicate task or resting state; event-related or block design.*
Design specifications	*Specify the number of blocks, trials or experimental units per session and/or subject, and specify the length of each trial or block (if trials are blocked) and interval between trials.*
Behavioral performance measures	*State number and/or type of variables recorded (e.g. correct button press, response time) and what statistics were used to establish that the subjects were performing the task as expected (e.g. mean, range, and/or standard deviation across subjects).*

Acquisition

Imaging type(s)	*Specify: functional, structural, diffusion, perfusion.*
Field strength	*Specify in Tesla*
Sequence & imaging parameters	*Specify the pulse sequence type (gradient echo, spin echo, etc.), imaging type (EPI, spiral, etc.), field of view, matrix size, slice thickness, orientation and TE/TR/flip angle.*
Area of acquisition	*State whether a whole brain scan was used OR define the area of acquisition, describing how the region was determined.*

Diffusion MRI ☐ Used ☐ Not used

Preprocessing

Preprocessing software	*Provide detail on software version and revision number and on specific parameters (model/functions, brain extraction, segmentation, smoothing kernel size, etc.).*
Normalization	*If data were normalized/standardized, describe the approach(es): specify linear or non-linear and define image types used for transformation OR indicate that data were not normalized and explain rationale for lack of normalization.*
Normalization template	*Describe the template used for normalization/transformation, specifying subject space or group standardized space (e.g. original Talairach, MNI305, ICBM152) OR indicate that the data were not normalized.*
Noise and artifact removal	*Describe your procedure(s) for artifact and structured noise removal, specifying motion parameters, tissue signals and physiological signals (heart rate, respiration).*

Volume censoring | *Define your software and/or method and criteria for volume censoring, and state the extent of such censoring.*

Statistical modeling & inference

Model type and settings | *Specify type (mass univariate, multivariate, RSA, predictive, etc.) and describe essential details of the model at the first and second levels (e.g. fixed, random or mixed effects; drift or auto-correlation).*

Effect(s) tested | *Define precise effect in terms of the task or stimulus conditions instead of psychological concepts and indicate whether ANOVA or factorial designs were used.*

Specify type of analysis: ☐ Whole brain ☐ ROI-based ☐ Both

Statistic type for inference | *Specify voxel-wise or cluster-wise and report all relevant parameters for cluster-wise methods.*

(See Eklund et al. 2016)

Correction | *Describe the type of correction and how it is obtained for multiple comparisons (e.g. FWE, FDR, permutation or Monte Carlo).*

Models & analysis

n/a	Involved in the study
☐	☐ Functional and/or effective connectivity
☐	☐ Graph analysis
☐	☐ Multivariate modeling or predictive analysis

Functional and/or effective connectivity | *Report the measures of dependence used and the model details (e.g. Pearson correlation, partial correlation, mutual information).*

Graph analysis | *Report the dependent variable and connectivity measure, specifying weighted graph or binarized graph, subject- or group-level, and the global and/or node summaries used (e.g. clustering coefficient, efficiency, etc.).*

Multivariate modeling and predictive analysis | *Specify independent variables, features extraction and dimension reduction, model, training and evaluation metrics.*
